# Multidimensional Comparisons Between Constrained ICA/IVA Algorithms for Multi-Subject fMRI Data Analysis

**DOI:** 10.1109/access.2026.3662260

**Published:** 2026-02-09

**Authors:** LUCAS GOIS, HANLU YANG, TRUNG VU, WEIXIN WANG, DENIS FANTINATO, ALINE NEVES, VINCE D. CALHOUN, TÜLAY ADALI

**Affiliations:** 1 Center for Engineering, Modeling and Applied Social Sciences, Federal University of ABC, Santo André 09280-560, Brazil; 2 Department of Computer Science and Electrical Engineering, University of Maryland, Baltimore County (UMBC), Baltimore, MD 21250, USA; 3 Department of Computer Engineering and Automation, Universidade Estadual de Campinas, Campinas 13083-852, Brazil; 4 Tri-Institutional Center for Translational Research in Neuroimaging and Data Science (TReNDS), Georgia State University, Georgia Institute of Technology, Emory University, Atlanta, GA 30303, USA

**Keywords:** fMRI, independent component analysis, independent vector analysis, joint blind source separation

## Abstract

Large-scale functional magnetic resonance imaging (fMRI) datasets provide exciting opportunities for understanding and improving brain health. Data-driven techniques such as independent component analysis (ICA) and independent vector analysis (IVA) have been attractive solutions for multi-subject fMRI analysis, as the extraction of functional connectivity networks is the key step in many studies. Constrained versions of ICA and IVA help significantly improve performance and interpretability, but their comparative advantages and the practical impact of their different formulations remain unclear. This work addresses this gap by conducting a comprehensive comparison of three state-of-the-art constrained algorithms: threshold-free constrained IVA (tf-cIVA), adaptive-reverse constrained IVA (ar-cIVA), and adaptive-reverse constrained ICA (ar-cEBM). These methods differ significantly in how they leverage the cross-subject information (joint processing of IVA versus the subject-wise approach of constrained ICA) and in their definitions of the closeness with the references (Lagrangian-based adaptive thresholding versus a threshold-free regularization term). We perform a multidimensional comparison among these methods using multiple metrics such as reproducibility, scalability, alignment with references, connectivity, and consistency on a multi-site fMRI dataset of 429 subjects. Our results reveal replicability across the three methods regarding their spatial correlation with the references and identification of biomarkers, as well as distinct trade-offs in other aspects: tf-cIVA excels in reproducibility and produces highly structured temporal functional network connectivity (FNC), making it a strong candidate for dynamic or connectivity-based analyses. Meanwhile, ar-cIVA demonstrates the greatest sensitivity to group differences in spatial FNC, suggesting its utility for identifying spatial biomarkers. Finally, ar-cEBM, via its subject-wise approach, offers superior computational scalability for large datasets. Surprisingly, despite not jointly modeling cross-subject information, ar-cEBM produces more stable spatial maps across subjects, suggesting its flexible density matching may be more critical for group consistency than the joint-processing framework itself. Therefore, besides providing a complete picture, the work provides practical guidance, indicating that the algorithm choice might depend on the specific research question.

## I. INTRODUCTION

Analysis of large-scale multi-subject functional magnetic resonance imaging (fMRI) datasets provides new opportunities to explore brain networks, identify biomarkers of mental disorders, and characterize group-level differences in brain functions [1], [2], [3], [4]. The increasing availability of neuroimaging datasets with large cohorts allows for the analysis of statistical differences in brain functions across both healthy populations and those affected by disorders, such as schizophrenia [5], autism [6], psychosis [7], and major depressive disorder [8]. However, analyzing large-scale fMRI data with advanced models poses major challenges such as achieving computational efficiency when handling high-dimensional data from many subjects, and effectively capturing their subject-specific variability [9], [10]. Summarizing the observed data through a latent factor model has proven attractive for multiple reasons, including direct interpretability of these factors while minimizing modeling assumptions. Data-driven methods have been widely employed in fMRI data analysis due to their ability to extract latent factors through simple generative models and are particularly attractive for not relying on model assumptions, but rather making use of different model-independent priors, such as independence or sparsity [11]. Among these, independent component analysis (ICA) is a popular blind source separation (BSS) technique that explores statistical independence among the underlying sources and has been successfully applied to fMRI data analysis [12], [13]. Group ICA (GICA) [14] extends ICA to multiple subjects by estimating components from a common subspace across subjects and using a back-reconstruction strategy, but it also limits the ability to preserve subject-specific variability. Independent vector analysis (IVA) generalizes ICA to multiple subjects [15] and presents a compelling alternative by leveraging inter-subject dependencies, allowing corresponding components across subjects to be aligned automatically. This property typically yields more consistent results and better captures the subject variability [10], [16]. Yet, IVA also introduces additional computational complexity due to the joint analysis over multiple subjects, and its performance can degrade as the number of subjects increases [17].

While ICA and IVA have been useful data-driven techniques for extracting functional connectivity networks from fMRI data, they do not take into account reliable prior information, which might help improve performance [5], [18]. To address this limitation, some methods have incorporated spatial priors or network templates into the decomposition process [19], [20]. In recent years, several methods have been proposed to incorporate prior information into ICA and IVA, showing substantial benefits by balancing the desirable data-driven aspects of ICA/IVA with those of model-based approaches. This prior information, also called *reference* in this context, is usually added to the cost function through spatial constraints [21], [22] and solved within a Lagrangian framework [7], [23]. Prior information can thus guide the estimation process toward physiologically meaningful sources and improve component alignment across subjects, thus improving interpretability, stability, and efficiency [24], [25]. However, many of these approaches depend on the choice of a threshold parameter that controls the closeness between an estimated component and its reference. Selecting this threshold can be a non-trivial problem, as small values may lead to weak guidance and suboptimal solutions. In contrast, overly large values prevent convergence to a meaningful solution since the corresponding constraint will not be satisfied [17], [23]. To address these limitations, two constrained IVA algorithms with different strategies for integrating prior information have been introduced [17]. The first, adaptive-reverse constrained IVA (ar-cIVA), alternates between conservative and assertive updates using a Lagrangian multiplier to dynamically adjust the correlation threshold between a component and its reference. The second, threshold-free constrained IVA (tf-cIVA), avoids threshold selection entirely by directly encouraging similarity between components and their references while discouraging correlations with non-corresponding templates.

In addition, constrained formulations of ICA have also been revisited for computational efficiency with large-scale datasets while also alleviating permutation ambiguity through the use of references. Prominent examples include the multiobjective optimization ICA with reference (MOO-ICAR) [25] and the constrained entropy-bound minimization (c-EBM) [26], both of which have proven effective in fMRI analysis. The latter is based on the entropy-bound minimization (EBM) procedure [27], which provides a flexible density matching strategy that more accurately captures the latent source distribution as well as a non-orthogonal general demixing matrix [26]. However, both of these approaches require the selection of a fixed constraint threshold. To address this, [28] proposed the adaptive-reverse constrained ICA (ar-cEBM), a new constrained ICA algorithm that employs an adaptive-reverse scheme similar to ar-cIVA for threshold adjustment throughout iterations, combining flexibility and interpretability.

Hence, these three algorithms: ar-cEBM operating on a per-subject basis, and ar-cIVA and tf-IVA processing all datasets together, provide a desirable balance between data-driven and model-based estimation, while also presenting distinct advantages and trade-offs. Constrained ICA approach, ar-cEBM, offers computational efficiency and retains subject-level details, however, it cannot explicitly leverage cross-subject dependencies. In contrast, the constrained IVA algorithms (ar-cIVA and tf-cIVA) explicitly model such dependencies, improving component correspondence across subjects at the cost of higher computational demand. Given these fundamental differences in joint-processing frameworks and constraint application, understanding their practical trade-offs is of great interest for advancing large-scale fMRI data studies. While they have been shown to offer advantages compared with the state-of-the-art, their performances for the most part are demonstrated on limited datasets, and more importantly, their performance with respect to each other has not been addressed.

A systematic comparison of these algorithms can thus reveal their relative strengths in key aspects of neuroimaging data analysis, including interpretability, reproducibility, replicability, sensitivity to subject variability, and computational efficiency. To this end, this work presents a comprehensive, multidimensional evaluation of ar-cIVA, tf-cIVA, and ar-cEBM. By analyzing their performance across multiple metrics and scenarios, we aim to provide insights into their behavior and practical guidelines for selecting suitable approaches for large-scale multi-subject fMRI studies, including applications such as population-level modeling, dynamic connectivity analysis, and subgroup identification. These recommendations will support researchers in choosing an algorithm that aligns with their specific research goals, such as prioritizing highly structured temporal connectivity, maximizing sensitivity to spatial group differences, or ensuring computational feasibility for large datasets. To the best of our knowledge, this is the first comprehensive multidimensional comparison between constrained ICA and constrained IVA algorithms on multi-subject fMRI datasets.

The remainder of this paper is structured as follows. Section II introduces the mathematical background and methodological framework, covering the ICA formulation by entropy-bound minimization, the general IVA model, and the proposed constraint formulations, including the adaptive-reverse and threshold-free schemes. Section III presents a detailed comparative analysis of the three algorithms across several evaluation dimensions. We first describe the set of metrics used in the comparison, encompassing reproducibility, both spatial and temporal functional network connectivity (FNC) analyses, and spatial map consistency. We then evaluate performance in terms of correlation with reference templates, connectivity patterns, statistical group comparisons, and overall modularity behavior. Additionally, we assess component activity and computational cost. Finally, Section IV summarizes the main findings and discusses the implications of the results for multi-subject fMRI analysis and future developments in constrained data-driven methods.

## II. METHOD

### A. ICA BY ENTROPY BOUND MINIMIZATION

ICA models the observed data from a single subject, $x(v)={\left[x_{1}(v),x_{2}(v),\ldots ,x_{N}(v)\right]}^{⊤}$, as a linear mixture of N statistically independent sources. For fMRI data, the index v typically represents a voxel, for v=1,…,V. The generative model is given by:

(1) x(v)=As(v),

where $A\in R^{N\times N}$ is an unknown mixing matrix and $s(v)={\left[s_{1}(v),s_{2}(v),\ldots ,s_{N}(v)\right]}^{⊤}$ are the statistically independent sources to be estimated. The objective of ICA is to determine a demixing matrix $W\in R^{N\times N}$ such that y(v)=Wx(v), where $y(v)={\left[y_{1}(v),y_{2}(v),\ldots ,y_{N}(v)\right]}^{⊤}$ are maximally independent and approximate s(v). This is achieved by using different properties of the signal, such as non-Gaussianity, nonstationarity, and sample dependence [12]. In this work, we focus on non-Gaussianity. Therefore, we assume the samples are independent and identically distributed (i.i.d.) and drop the sample index v for notational convenience. The standard approach is to minimize the mutual information among the N estimated sources, $y_{n}$ for n=1,…,N, which can be expressed as [12]:

(2) $${𝒥}_{\text{ICA}}(W)=\sum_{n=1}^{N}ℋ\left(y_{n}\right)-\text{log}|\text{det}\;W|-ℋ(x),$$

where $ℋ\left(y_{n}\right)=-E\left[\text{log}\;p_{y_{n}}\left(y_{n}\right)\right]$ is the (differential) entropy of $y_{n}$, estimated using its probability density function (PDF), $p_{y_{n}}\left(y_{n}\right)$. The last term, ℋ(x), is the entropy of x, which is a constant with respect to W.

Many ICA algorithms estimate the entropy by assuming a specific parametric model for the source distributions or by using a fixed nonlinear function to approximate the PDFs of the latent sources. While often effective, these methods are suboptimal if the assumed model is a poor match for the true latent source distributions.

Entropy bound minimization (ICA-EBM) offers a more flexible and robust solution for ICA by avoiding such restrictive assumptions by calculating several maximum entropy bounds and then selecting the tightest bound as the most accurate estimate of the entropy [27]. This process allows EBM to effectively perform density matching for a wide variety of distributions using only a finite set of pre-defined measuring functions, $G_{b(n)}$, for b=1,…,B. The EBM has proven effective for fMRI analysis by offering a flexible, stable, and efficient solution [11], [29].

### B. IVA FORMULATION

IVA is a JBSS method that extends ICA to simultaneously analyze multiple datasets by leveraging statistical dependence across corresponding sources. Assume we have K datasets ${\left\{x^{\left[k\right]}(v)\right\}}_{k=1}^{K}$, each modeled as:

(3) $$x^{\left[k\right]}(v)=A^{\left[k\right]}s^{\left[k\right]}(v),$$

where $s^{\left[k\right]}(v)={\left[s_{1}^{\left[k\right]}(v),s_{2}^{\left[k\right]}(v),\ldots ,s_{N}^{\left[k\right]}(v)\right]}^{⊤}$ are source vectors for the kth dataset. IVA introduces source component vectors (SCVs) by grouping the nth source across all K datasets, which is modeled as a random vector process:

(4) $$s_{n}(v)={\left[s_{n}^{\left[1\right]}(v),s_{n}^{\left[2\right]}(v),\ldots ,s_{n}^{\left[K\right]}(v)\right]}^{⊤}.$$

The goal of IVA is to estimate a set of demixing matrices, $𝒲={\left\{W^{\left[k\right]}\right\}}_{k=1}^{K}$, so the estimated sources are given by $y^{\left[k\right]}(v)=W^{\left[k\right]}x^{\left[k\right]}(v)$. The nth estimated SCV is given by $y_{n}(v)={\left[y_{n}^{\left[1\right]}(v),\ldots ,y_{n}^{\left[K\right]}(v)\right]}^{⊤}$. As in the ICA case, we utilize non-Gaussianity. Hence, we assume that the sources are i.i.d. and treat them as random variables. For simplicity, we drop the sample index v in the remainder of the paper.

A fundamental assumption in IVA is that the SCVs are mutually independent. This is achieved by minimizing the mutual information among the N SCVs, leading to the cost function [12]:

(5) $${𝒥}_{\text{IVA}}(𝒲)≜\sum_{n=1}^{N}ℋ\left(y_{n}\right)-\sum_{k=1}^{K}\text{log}\left|\text{det}\;W^{\left[k\right]}\right|.$$

To illustrate how IVA explicitly models statistical dependence across datasets, we can rewrite (5) as

(6) $${𝒥}_{\text{IVA}}(𝒲)=\sum_{n=1}^{N}\left(\sum_{k=1}^{K}ℋ\left(y_{n}^{\left[k\right]}\right)-ℐ\left(y_{n}\right)\right)-\sum_{k=1}^{K}\text{log}\left|\text{det}\;W^{\left[k\right]}\right|,$$

where the term $\sum_{n=1}^{N}ℐ\left(y_{n}\right)$ is the sum of mutual information within each SCV, which takes the diversity across datasets into account. By minimizing (6), we maximize the mutual information among components of an SCV [12]. Additionally, IVA models each SCV using a multivariate PDF, which leverages any statistical dependence across datasets through the chosen SCV model [30]. In this work, we use the version of IVA that models the SCVs using a multivariate Gaussian distribution, referred to as IVA-G [9], which exploits second-order statistics (SOS). This approach provides a computationally efficient and effective solution for fMRI analysis [17]. For convenience, we will refer to IVA-G as simply IVA in the remainder of this paper.

### C. CONSTRAINED FORMULATIONS

While ICA and IVA are efficient data-driven methods, their performance can be significantly enhanced by incorporating prior information into their formulation. This practice is valuable in fMRI analysis, since unconstrained algorithms can sometimes converge to solutions that are difficult to interpret or are dominated by noise and artifacts. By introducing constraints, we can guide the algorithm towards meaningful solutions, thus improving the interpretability, stability, and alignment of the estimated components.

In this work, the set of reference signals, ${\left\{r_{n}\right\}}_{n=1}^{M}\subset R^{V}(M\leq N)$, are spatial templates of the brain networks. The number of references, M, is chosen as less than or equal to the total number of components to be estimated, N. If the number of components N>M, it allows for the estimation of “free” components that can capture unexpected signals or artifacts. The goal of the constraint is to ensure that for each reference $r_{n}$, the algorithm estimates a corresponding component, $y_{n}$, that is highly similar to it. Furthermore, by leveraging information from the references, the algorithms indirectly exploit higher-order statistics (HOS) [17].

All three constraint formulations evaluated in this paper rely on the absolute Pearson correlation to quantify the similarity between two signals a and b:

(7) $$\epsilon \left(a,b\right)=\frac{\left|a^{⊤}b\right|}{\left\|a\right\|\left\|b\right\|}\in \left[0,1\right].$$

The idea of constrained methods is to ensure that reference, $r_{n}$, is more similar to its corresponding estimated component, $y_{n}^{\left[k\right]}$, than to any other $y_{m}^{\left[k\right]}(m\neq n)$ [7]. This is often implemented by selecting the value of a pre-defined threshold parameter, ρ, that defines the required closeness between the estimated components and the reference signals [31]:

(8) $$\epsilon \left(r_{n},y_{n}^{\left[k\right]}\right)\geq \rho >\epsilon \left(r_{n},y_{m}^{\left[k\right]}\right),\;\forall m\neq n.$$

However, selecting an appropriate value for ρ is a challenge in practice. Small threshold values can lead to suboptimal solutions, whereas a value too high may prevent the constraint from being satisfied [17]. While adaptive schemes for selecting thresholds exist [32], they can also sometimes be suboptimal. Two recent algorithms provide attractive solutions for this problem: either determining the threshold via adaptive-reverse schemes, such as ar-cIVA [17] and ar-cEBM [28], or avoiding thresholds altogether, as seen in tf-cIVA [17]. The following subsections will detail the three distinct methods evaluated in this paper for incorporating constraints.

#### 1) ADAPTIVE-REVERSE CONSTRAINED IVA (AR-CIVA)

Proposed in [17], ar-cIVA addresses the limitations of fixed thresholding by adaptively selecting a threshold $\rho_{n}^{\left[k\right]}$ for each component n and each subject k, allowing it to account for subject variability across components. The optimization problem is formulated as:

(9) $$\underset{𝒲}{\text{min}}\;{𝒥}_{\text{IVA}}\left(𝒲\right)\;\text{s.t.}\;\epsilon \left(r_{n},y_{n}^{\left[k\right]}\right)\geq \rho_{n}^{\left[k\right]},\;\forall n,k.$$

The threshold $\rho_{n}^{\left[k\right]}$ is selected from a predefined set of values P by alternating between two schemes:

(10) $$\rho_{n}^{\left[k\right]}=\text{arg}\;\text{min}\left\{\rho \in P|\rho >\epsilon \left(r_{n},y_{n}^{\left[k\right]}\right)\right\},\;\text{and}$$

(11) $$\rho_{n}^{\left[k\right]}=\text{arg}\;\text{max}\left\{\rho \in P|\rho \leq \epsilon \left(r_{n},y_{n}^{\left[k\right]}\right)\right\}.$$

The first scheme (10) is more assertive and selects the smallest value that does not satisfy the constraint, which forces the similarity to increase after each iteration. The second scheme (11) presents a more conservative behavior by choosing the largest value that satisfies the constraint. Thus, by switching between both schemes at each iteration, it is possible to recover the desired threshold value.

The optimization of (9) is solved by using the augmented Lagrangian method combined with a decoupling method [17]. This decoupling approach simplifies the procedure by enabling sequential updates of each row of the demixing matrices, $w_{n}^{\left[k\right]}$, effectively transforming the matrix problem into a series of vector optimization problems. The augmented Lagrangian method incorporates the constraints into the IVA cost function, resulting in the following equation [17]:

(12) $${ℒ}_{\gamma ,\rho }(𝒲,\mu )={𝒥}_{\text{IVA}}(𝒲)+\frac{1}{2\gamma }\sum_{n=1}^{M}\sum_{k=1}^{K}\left[{\left(\text{max}\left(0,\mu_{n}^{\left[k\right]}+\gamma \left(\rho_{n}^{\left[k\right]}-\epsilon \left(r_{n},y_{n}^{\left[k\right]}\right)\right)\right)\right)}^{2}\right.\left.-{\left(\mu_{n}^{\left[k\right]}\right)}^{2}\right],$$

where $\mu \in R^{M\times K}$ are the Lagrange multipliers and γ>0 is a scalar penalty parameter [33]. The adaptive-reverse scheme works through the Lagrange multiplier $\mu_{n}^{\left[k\right]}$, which verifies if each constraint is satisfied. When $\mu_{n}^{\left[k\right]}$ increases beyond a predefined threshold $\mu_{\text{max}}$, it switches to the conservative scheme (11). On the other hand, when $\mu_{n}^{\left[k\right]}$ approaches 0, the algorithm returns to the assertive scheme (10) [17].

#### 2) THRESHOLD-FREE CONSTRAINED IVA (TF-CIVA)

As an alternative to selecting threshold values, tf-cIVA was proposed as a method that avoids thresholds entirely by introducing a novel regularization term [17]. This term leverages the similarity measure between a reference $r_{n}$ and its corresponding estimated component $y_{n}^{\left[k\right]}$, while also discouraging similarity between $r_{n}$ and any other estimated component $y_{m}^{\left[k\right]}$ (where m≠n), and is defined as:

(13) $${𝒥}_{\text{ref}}\left(𝒲\right)=\sum_{n=1}^{M}\sum_{k=1}^{K}\left(\sum_{m=1,m\neq n}^{M}\epsilon^{2}\left(r_{n},y_{m}^{\left[k\right]}\right)-\epsilon^{2}\left(r_{n},y_{n}^{\left[k\right]}\right)\right).$$

The cost function of tf-cIVA is then a weighted sum of the IVA cost and this regularization term:

(14) $${ℒ}_{\text{tf-cIVA}}\left(𝒲\right)={𝒥}_{\text{IVA}}\left(𝒲\right)+\frac{\lambda }{2}{𝒥}_{\text{ref}}\left(𝒲\right),$$

where λ>0 is a regularization parameter that controls the trade-off between the IVA cost (maximizing the independence among the SCVs) and the regularization term (promoting alignment with the references). Furthermore, by also including the cross-component similarity in the objective function, this method promotes a solution in which there is only one independent component that is closest to each reference [17].

#### 3) ADAPTIVE-REVERSE CONSTRAINED ICA (AR-CEBM)

Proposed in [28], ar-cEBM extends the ICA-EBM framework by incorporating spatial constraints and the adaptive-reverse scheme for dynamic threshold adjustment. Unlike the IVA-based algorithms that jointly estimate shared components across subjects, ar-cEBM processes each subject independently, which allows for greater scalability but limits the direct modeling of inter-subject dependencies. This method leverages EBM’s flexibility in modeling subject-specific characteristics, while aligning the estimated components through the use of constraints.

A key feature in constrained ICA is the decoupling method, as it allows constraints to be applied to individual demixing vectors (or components) separately, which is particularly important since prior information about the entire demixing matrix is usually not available [31]. By using the entropy estimated by the tightest maximum entropy bound and applying the decoupling, the constrained optimization problem is formulated to minimize the ICA-EBM cost function [27], ${𝒥}_{\text{EBM}}\left(w_{n}\right)$, for each subject as:

(15) $$\underset{w_{n}}{\text{min}\;}{𝒥}_{\text{EBM}}\left(w_{n}\right)\;\text{s.t.}\;\epsilon \left(r_{n},y_{n}\right)\geq \rho_{n},\;\forall n\leq M.$$

Similar to ar-cIVA, the constrained optimization problem (15) is solved for each subject using the augmented Lagrangian method, iteratively updating the demixing matrix W and the Lagrange multipliers $\mu_{n}$ [28]. Notably, because the optimization is subject-wise, the threshold $\rho_{n}$ is indexed only by the component. The adaptive-reverse scheme adjusts $\rho_{n}$ during the independent optimization of each subject, preserving the subject-specific characteristics while still leveraging the guidance of the references.

## III. MULTI-SUBJECT fMRI DATA ANALYSIS

### A. COMPARISON FRAMEWORK

In this section, we evaluate how the three distinct constrained JBSS algorithms (tf-cIVA, ar-cIVA, and ar-cEBM) perform in capturing the complex patterns of functional brain organization. While all three methods leverage spatial priors to guide component estimation, their main differences in PDF modeling and constraint application result in distinct trade-offs. The IVA-based methods, tf-cIVA and ar-cIVA, perform a joint BSS across all subjects that exploits statistical dependence across datasets. Due to the IVA-G model, these methods make use of SOS, but also incorporate HOS indirectly through the spatial constraints. Their primary distinction lies in the constraint application: tf-cIVA utilizes a threshold-free regularization term that encourages similarity to the corresponding reference and dissimilarity to others, while ar-cIVA uses an adaptive-reverse scheme to select correlation thresholds dynamically. In contrast, ar-cEBM operates on a subject-wise level, applying constrained ICA independently to each individual, enhancing its computational scalability. It shares the adaptive-reverse thresholding scheme with ar-cIVA but applies it at the individual subject level. Furthermore, ar-cEBM directly exploits HOS by using the flexible density matching of the EBM framework. These differences between the three constrained methods result in expected trade-offs when dealing with multisubject fMRI data analysis. To quantify algorithmic performance, we employed a set of complementary metrics designed to capture multiple dimensions of brain network organization and algorithm behavior:

- **Cross-joint Inter-Symbol Interference (Cross-joint ISI)**: Since the algorithms are initialized randomly, this metric is important for evaluating the algorithms’ reproducibility [34]. It measures the consistency of estimated components across multiple independent runs of an algorithm. A lower cross-joint ISI value indicates that multiple runs from one algorithm converge to a similar solution regardless of its starting point, which implies higher stability.
- **Similarity with the references:** Calculated using the Pearson correlation between the spatial maps and their corresponding reference. This measure evaluates the alignment of the spatial maps with the templates that each constrained method achieved.
- **Temporal Functional Network Connectivity (Temporal FNC):** Temporal FNC is computed as the Pearson correlation between the subject-specific time courses of the estimated independent components. It provides a measure of the temporal relationships between different brain networks.
- **Spatial Functional Network Connectivity (Spatial FNC):** Measured as the Pearson correlation between component spatial maps, spatial FNC shows the spatial relationships and potential overlap between different networks, reflecting their spatial arrangement.
- **Modularity:** This graph-theory metric quantifies how well a network separates into distinct communities (modules). A high modularity score, calculated on an FNC matrix, indicates a well-organized network with strong within-module connectivity and sparse connectivity between modules.
- **One-sample t-maps:** By performing voxel-wise one-sample t-tests, we evaluate the quality and consistency of the spatial maps across subjects. Higher t-values indicate lower variability across subjects.

### B. DATA AND IMPLEMENTATION

The dataset used in this study was obtained from the Bipolar-Schizophrenia Network on Intermediate Phenotypes 2 (BSNIP-2) project [35], a multi-site neuroimaging initiative designed to identify biomarkers of psychosis. BSNIP-2 includes resting-state functional MRI (rs-fMRI) data collected across multiple clinical sites from healthy controls and individuals with diagnoses across the psychosis spectrum.

For the purposes of this work, we restricted the dataset to two groups: healthy controls (HC) and patients with schizophrenia (SZ). After applying these criteria, the final sample comprised a total of 429 subjects: 228 HC and 201 SZ. This cohort was drawn from three sites: Chicago (58 HC and 58 SZ), Dallas (59 HC and 56 SZ), and Hartford (111 HC and 87 SZ).

The rs-fMRI data were captured using a 3-Tesla scanner with TE = 30 ms, TR = 2 s, flip angle = 60°, acquisition matrix = 64 × 64 mm, and the voxel size = 3.4 × 3.4 × 5 mm^3^. The data underwent a series of standard preprocessing steps, including discarding the first six time points of each scan to address the T1-effect, slice-time correction, motion correction, spatial normalization to the Montreal Neurological Institute EPI standard, and smoothing using a Gaussian kernel with a full width at half maximum (FWHM = 6 mm). Each subject image was masked to exclude non-brain voxels and flattened, resulting in an observation vector of V=57878 voxels for each of the remaining T=224 time points.

We utilized a standard set of M=53 resting-state network (RSN) templates derived from the NeuroMark pipeline [21]. These references are well-established templates representing common brain networks and are categorized into seven functional domains: sub-cortical (SC), auditory (AUD), sensorimotor (MOT), visual (VIS), cognitive-control (CC), default-mode (DM), and cerebellar (CB). Prior to running the constrained methods, we applied subject-level principal component analysis (PCA) to reduce the dimensionality of the data in the temporal dimension. To define the number of source components, we used the entropy-rate-based model order selection by finite memory length (ER-FM) and autoregressive model (ER-AR) on the fMRI data [36]. The model order found in this analysis with ER-FM is 73.149 ± 12.587, whereas it is 76.677 ± 13.484 with ER-AR. Based on these numbers, we estimated a total of N=85 components for each subject in all algorithms in order to include potential artifacts or unexpected sources of neural activity.

The three algorithms were implemented using parameters consistent with their original publications for fMRI analysis. All methods started from the same random initializations, and the learning process was terminated when the maximum change in the demixing vectors between iterations fell below a threshold of $\epsilon =10^{-6}$. An adaptive step size was used, which decayed by a factor of 0.95 if the objective function failed to decrease. For the two algorithms employing the adaptive-reverse scheme, ar-cIVA and ar-cEBM, the hyperparameters were: a predefined set of thresholds 𝒫={0.01,0.02,…,0.99}, a penalty parameter γ=100, and a Lagrange multiplier threshold value of $\mu_{\text{max}}=1$. For the threshold-free method, tf-cIVA, the regularization parameter was set to λ=100, as recommended for real fMRI data analysis in the original study [17]. To manage the computational load of the joint-processing algorithms and to assess site consistency, all three methods were run independently on the data from each of the three sites. The resulting components were then concatenated for the final 429-subject group-level analyses. We also verified that the main findings reported in this paper (such as group differences and spatial map consistency) were observable within each site individually, ensuring the concatenated results are replicable and not driven by site-specific effects.

### C. REPRODUCIBILITY

Given that the algorithms under evaluation rely on random initializations, an important aspect of their performance is reproducibility, i.e., their ability to produce consistent results across multiple independent runs using the same data [34]. Consistent results across multiple runs provide evidence that the solution is not driven by the algorithm’s initialization or local minima. To quantify reproducibility, we evaluated their stability using cross-joint ISI [37]. This is an effective and efficient metric that builds upon the standard inter-symbol interference (ISI) used in blind source separation [38], [39].

The joint-ISI extends the standard ISI to evaluate the overall separation performance across K datasets, assuming that the ground-truth mixing matrices $A^{\left[k\right]}$ are known [9]. Let the global demixing-mixing matrix for the kth subject be $G^{\left[k\right]}=W^{\left[k\right]}A^{\left[k\right]}$, with the (m,n) entry being $G_{m,n}^{\left[k\right]}$. The joint-ISI is defined as the ISI applied to the mean absolute global matrix of all subjects, $G=\frac{1}{K}\sum_{k=1}^{K}\left|G^{\left[k\right]}\right|$, thus: $\text{joint-ISI}\left(G^{\left[1\right]},\ldots ,G^{\left[K\right]}\right)=\text{ISI}(G)$. The ISI value for a single global matrix G is calculated as:

(16) $$\text{ISI}(G)=\frac{\left[\sum_{i=1}^{N}\left(\frac{\sum_{j=1}^{N}\left|G_{ij}\right|}{{\text{max}}_{p}\left|G_{ip}\right|}-1\right)+\sum_{j=1}^{N}\left(\frac{\sum_{i=1}^{N}\left|G_{ij}\right|}{{\text{max}}_{p}\left|G_{pj}\right|}-1\right)\right]}{2N(N-1)}.$$

The cross-joint ISI measures the consistency across multiple independent R runs of an algorithm. Let $W_{i}^{\left[k\right]}$ and $W_{j}^{\left[k\right]}$ be the estimated demixing matrices for the kth subject from runs i and j, respectively. The performance matrix between these runs for subject k is $P_{ij}^{\left[k\right]}=A_{i}^{\left[k\right]}W_{j}^{\left[k\right]}={\left(W_{i}^{\left[k\right]}\right)}^{-1}W_{j}^{\left[k\right]}$. The cross-joint-ISI between runs i and j is then defined as the joint-ISI computed using these performance matrices:

(17) $${\text{cross-joint-ISI}}_{ij}\left({\left\{W_{r}^{\left[k\right]}\right\}}_{r=1,k=1}^{R,K}\right)=\text{joint-ISI}\left(P_{ij}^{\left[1\right]},\ldots ,P_{ij}^{\left[K\right]}\right).$$

The overall cross-joint ISI for the ith run is calculated as the average of these pairwise values between run i and all other runs (j≠i):

(18) $${\text{cross-joint-ISI}}_{i}=\frac{1}{R-1}\sum_{j=1,j\neq i}^{R}{\text{cross-joint-ISI}}_{ij}.$$

A value close to zero indicates high reproducibility, meaning the algorithm consistently converges to similar solutions regardless of initialization. We performed this analysis for R=20 runs, and the results are summarized in Table 1.

The results show that the tf-cIVA algorithm demonstrates higher reproducibility compared with the other two methods. Its cross-ISI values are the lowest by a significant margin, and its partial cross-ISI, which considers only the constrained components, is low and stable. This high stability is likely a direct consequence of its threshold-free regularization term in the cost function that guides the constrained components towards the reference signals. This feature creates a well-defined structure that is less sensitive to the random starting point and ensures the algorithm converges to similar solutions on each run. Furthermore, tf-cIVA offers a computational advantage compared with other algorithms, as it consistently converges to similar solutions regardless of the random starting point. Thus, there is no need to search for the best run across a large number of independent runs.

Following tf-cIVA, ar-cIVA also shows strong reproducibility, with cross-ISI values that are only moderately higher. Its IVA framework helps enforce a consistent group structure, contributing to its stability. In contrast, ar-cEBM is the least stable of the three, with significantly higher cross-ISI values. This is an expected trade-off of its subject-wise approach since the free components are subject to the permutation ambiguity. Looking only at the constrained components, both adaptive-reverse scheme algorithms show higher partial cross-ISI, indicating that their threshold selection method is less stable in comparison to the threshold-free method, and that the threshold handling aspect seems to be an important determinant of reproducibility.

### D. ALIGNMENT WITH THE REFERENCES

**FIGURE 1.**
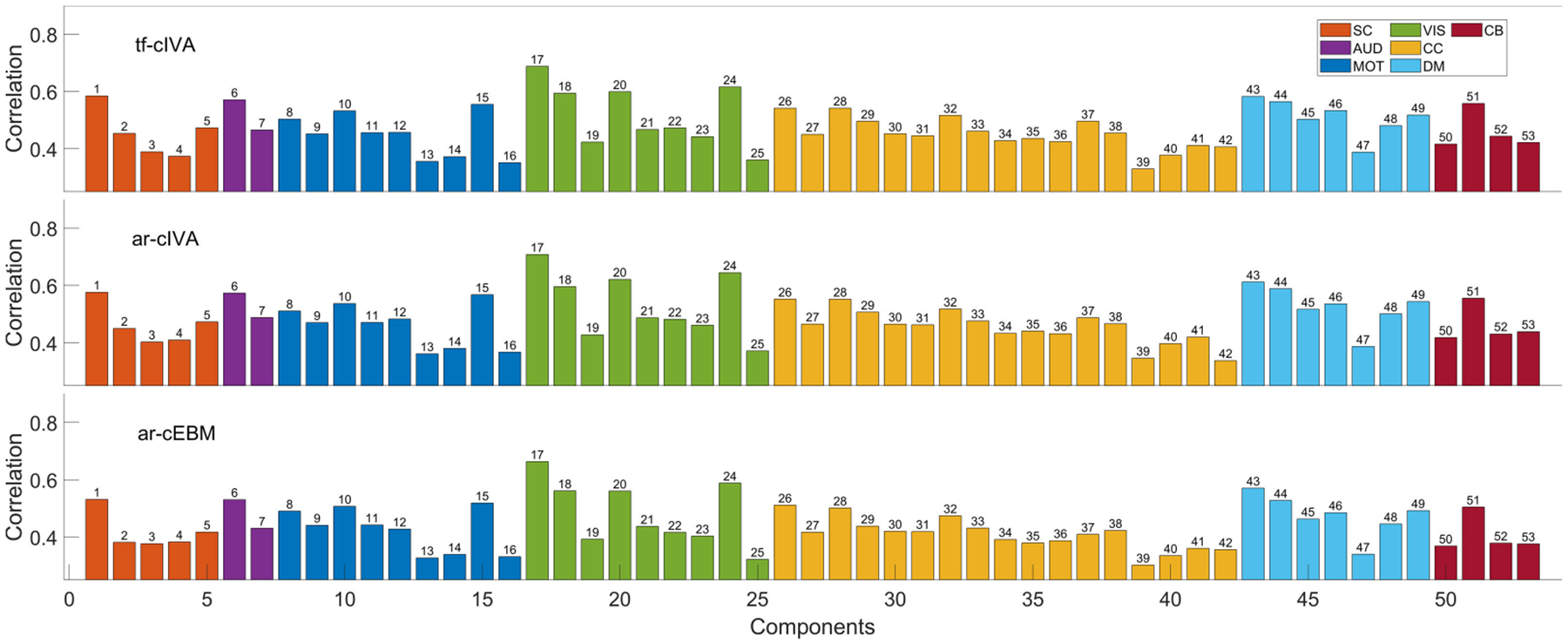
Mean spatial correlation between estimated components and their corresponding NeuroMark references. The bars represent the average correlation across all 429 subjects for each of the 53 components. The results display that the three different methods achieve a similar correlation profile across components, indicating that all three algorithms achieve comparable estimation accuracy.

A direct metric for evaluating the constrained algorithms is the spatial similarity of the estimated components with the provided reference templates. We began by computing the mean Pearson correlation between each of the 53 constrained components and its corresponding NeuroMark reference. As shown in Figure 1, it is interesting to notice that all three algorithms exhibit a similar correlation profile across the components (i.e., the correlation values rise and fall in conjunction for the same components), indicating that the methods generally agree on which references are more or less correlated. Quantitatively, ar-cIVA achieved the highest correlation for 36 out of 53 constrained components, while tf-cIVA attained the highest correlation of the 17 that remained.

To investigate group differences, we compared the spatial correlation distributions of HC and SZ for each component using two different statistical tests: the two-sample t-test and the two-sample Kolmogorov-Smirnov (KS) test, both at a significance level of 0.05. We employed both tests to capture different types of group differences. The t-test assesses whether the mean correlation value is significantly different between the two groups, whereas the KS test is sensitive to any difference in the overall shape of the two distributions. The KS statistic, D, is defined as the maximum absolute difference between the cumulative distribution functions (CDFs) of the correlation values for the two groups:

(19) $$D=\underset{x}{\text{sup}}\left|F_{HC}(x)-F_{SZ}(x)\right|,$$

where $F_{HC}(x)$ and $F_{SZ}(x)$ are the CDFs of HC and SZ groups, respectively.

**FIGURE 2.**
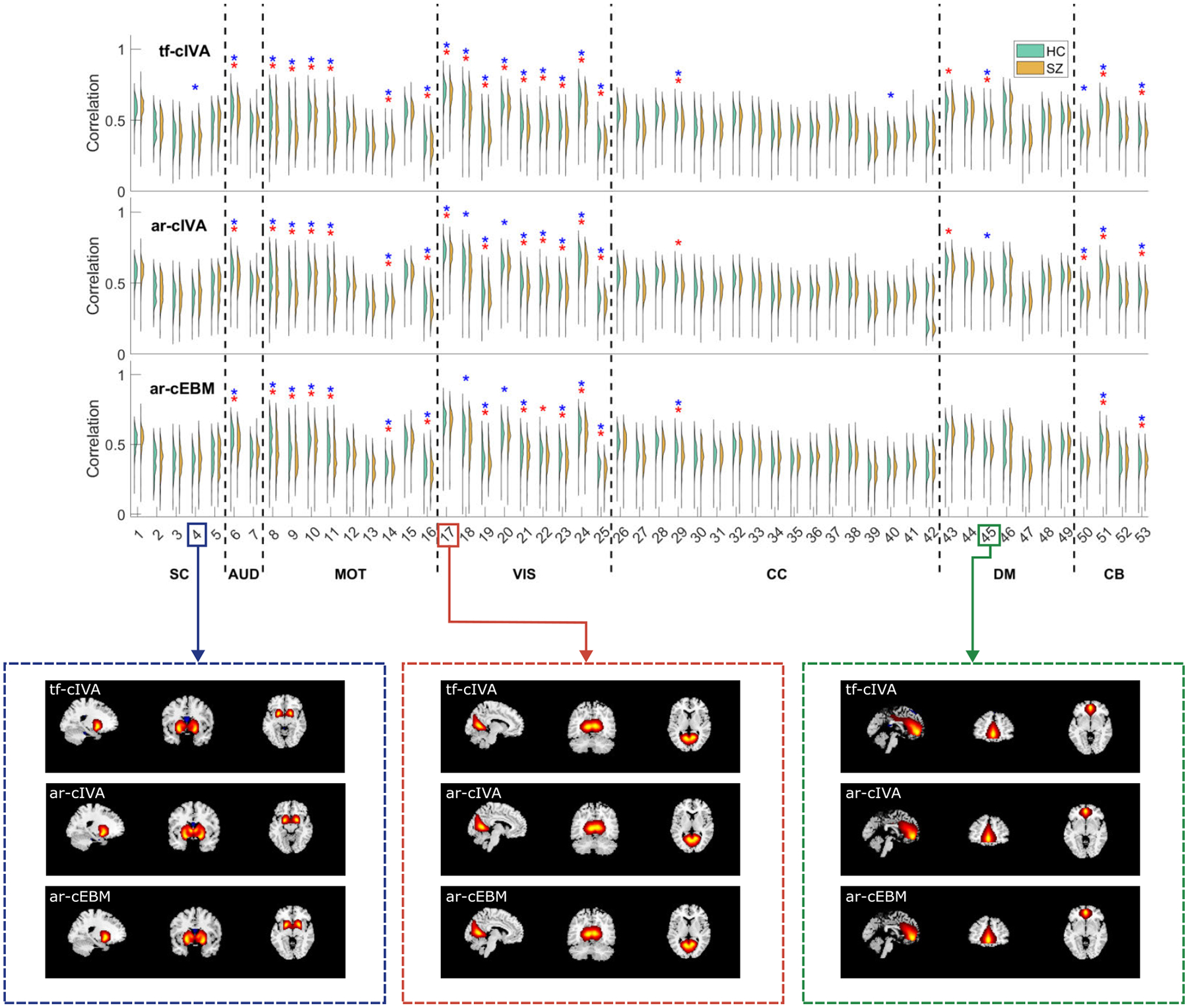
Distribution of spatial correlations between estimated components and references, showing the distributions for healthy controls (HC) and schizophrenia (SZ) patients. Stars denote components with statistically significant group differences, as determined by both the two-sample t-test (p<0.05) in blue stars and the Kolmogorov-Smirnov (KS) test (α<0.05) in red stars. The IVA-based methods, tf-cIVA and ar-cIVA, identify more components with significant group differences in VIS, DM, and CB networks, thus resulting in greater sensitivity to biomarkers in these activation regions. For example, the spatial maps from SC, VIS, and DM show different results regarding the statistical test for the three algorithms.

The violin plots in Figure 2 show the shapes of correlation value distributions for each constrained component, where HC is in green, and SZ is in yellow. Above each pair of distributions, we indicate if they reject the null hypothesis for the t-test (blue stars) and the KS test (red stars). We observe that most of the meaningful components, i.e., those with stars, are concentrated in the MOT and VIS domains for the three algorithms, indicating that they were able to detect putative biomarkers consistently in these domains. Observing the patterns across algorithms, we notice that some domains, such as AUD and MOT, exhibit high replicability across all three methods. In contrast, the VIS, DM, and CB networks display some differences depending on the algorithm, with the IVA-based methods presenting more commonalities with each other. Overall, the violin plots show that tf-cIVA is the most sensitive in detecting group differences in spatial correlation with the references, identifying more components with significantly different distributions for both statistical tests compared with ar-cIVA and ar-cEBM. In total, tf-cIVA identified 23 meaningful components using the t-test and 21 using the KS test, ar-cIVA found 20 via t-test and 19 via KS test, whereas ar-cEBM resulted in 17 using the t-test and 16 using the KS test. The IVA-based methods were more sensitive than ar-cEBM, in particular in the VIS, DM, and CB networks. This suggests that, when analyzing the alignment of the estimated components with the references, leveraging the dependence across subjects can result in more significant group differences.

### E. CONNECTIVITY

#### 1) TEMPORAL FNCS

To evaluate the connectivity between the estimated brain networks, we calculated the temporal FNC for each subject. First, each subject’s time courses of each algorithm were detrended and despiked. Subsequently, pairwise Pearson’s correlation coefficients were computed between all pairs of time courses, resulting in a symmetric FNC matrix for each subject [40]. These correlation values were then Fisher z-transformed for subsequent statistical analyses. Additionally, the FNCs can be seen as an adjacency matrix, where each brain network is a node and the edges are given by their connectivity. To evaluate the connectivity structure of each algorithm, we calculated modularity as an effective graph-theory metric [41]. This metric quantifies the degree to which a network can be subdivided into non-overlapping groups of nodes (modules) such that connections within modules are dense and connections between modules are sparse. To calculate modularity, all the negative values and self-connections were first removed from the FNC matrices [42]. The remaining connections were then thresholded and binarized before computing the modularity, with the edges below the threshold having a value of 0 and those above having a value of 1. We define the percentage of remaining edges as link density, which increases as the threshold decreases. To avoid very sparse graphs or those with too high link density, we kept the link density between 20 and 70% [11]. We used an optimization algorithm [43] to find the partition that maximizes the modularity index Q, defined as [41]:

(20) $$Q=\frac{1}{L}\sum_{i,j\in N}\left[{𝒜}_{ij}-\frac{d_{i}d_{j}}{L}\right]\delta \left(m_{i},m_{j}\right),$$

where L is the total number of edges in the thresholded graph, ${𝒜}_{ij}$ is the adjacency matrix (1 if an edge exists between node i and j, 0 otherwise), $d_{i}$ is the degree of node $i,m_{i}$ is the module assignment of node i, and $\delta \left(m_{i},m_{j}\right)$ is 1 if nodes i and j are in the same module and 0 otherwise. A higher Q value indicates a stronger modular structure within the FNC matrix [42].

**FIGURE 3.**
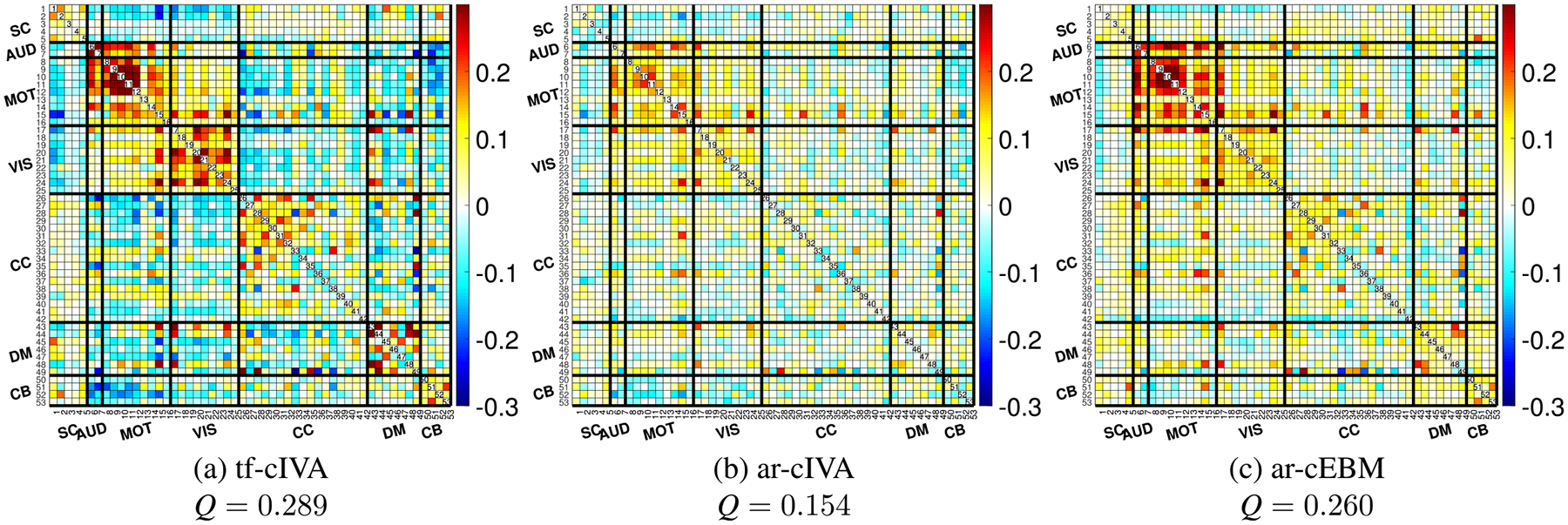
Mean temporal FNC matrices, averaged across all 429 subjects. Panels show the results for (a) tf-cIVA, (b) ar-cIVA, and (c) ar-cEBM. The matrices have distinct connectivity patterns, with tf-cIVA showing the most structured network, characterized by strong positive correlations within known functional domains (e.g., MOT, VIS) and clear anticorrelations between MOT/VIS and the DM network. This clear structure is quantitatively reflected in its higher modularity value.

The mean temporal FNC matrices, averaged across all subjects and then inverse Fisher z-transformed for visualization, are shown in Figure 3. While they agree in general, the temporal correlation structure obtained by each algorithm shows certain differences. In Figure 3(a), the FNC matrix from tf-cIVA exhibits a clear structure, showing positive correlations within MOT and VIS domains, and anticorrelations between MOT/VIS and DM networks, consistent with previous observations [40]. This visual structure is consistent with its higher temporal modularity score (Q=0.289) compared with the other algorithms, likely reflecting the influence of its regularization term on the estimated time courses. This result is in contrast with Figure 3(b), where ar-cIVA did not present such a clear block structure in the temporal FNC, resulting in a modularity value of 0.154. Finally, in Figure 3(c), ar-cEBM presents a positive block structure in the MOT and VIS domains, which reflects its modularity value (0.260). Overall, the constrained algorithms with the adaptive-reverse scheme do not present the expected anticorrelation between domains.

**FIGURE 4.**
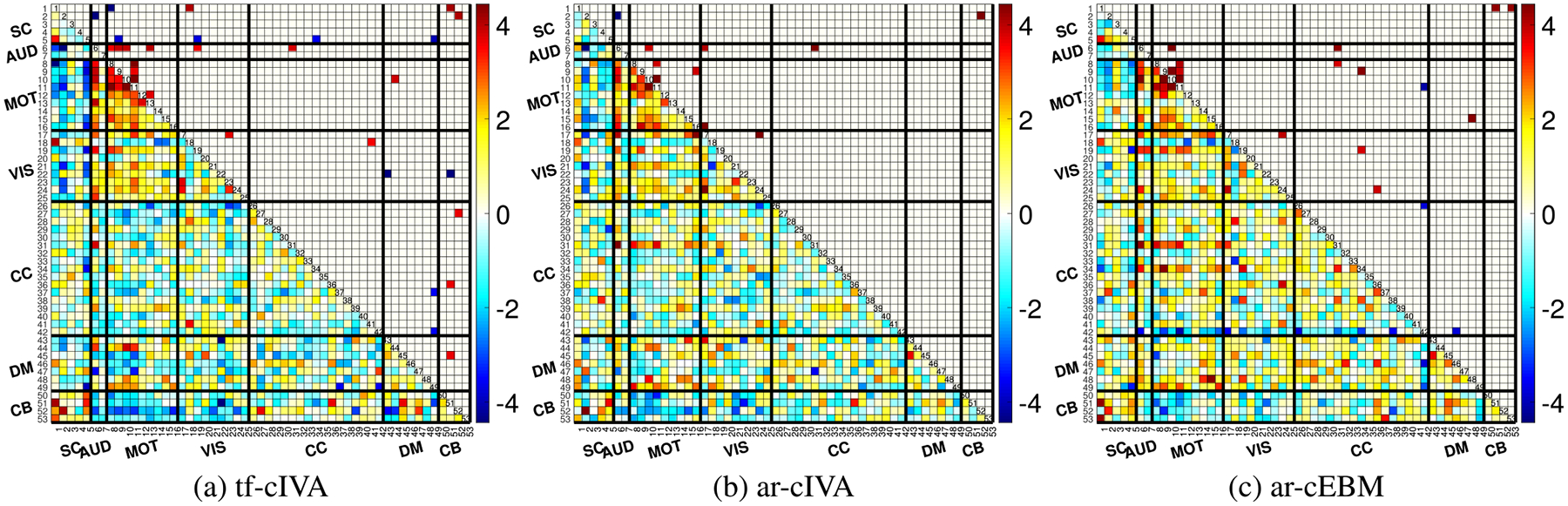
Two-sample t-test results (p<0.05, FDR corrected) for temporal FNCs comparing Healthy Controls (HC) and Schizophrenia (SZ) patients. Panels (a), (b), and (c) show the results for tf-cIVA, ar-cIVA, and ar-cEBM, respectively. The lower diagonal shows all t-statistics, and the upper diagonal shows significant differences after FDR correction. Although all methods show widespread differences, tf-cIVA identifies the largest number of significant group differences that survive FDR correction, as quantified in Table 3.

To investigate group-level differences in temporal FNCs, we performed two-sample t-tests comparing the Fisher z-transformed FNC matrices of HC and SZ groups for each algorithm. To account for multiple comparisons across all connectivity pairs, we applied a False Discovery Rate (FDR) correction to the resulting p-values, with a significance level of p<0.05 [44]. The results of these t-tests are shown in Figure 4, where the lower diagonal shows all t-statistics and the upper diagonal presents significant differences after FDR correction.

When comparing the number of significant group differences surviving FDR, tf-cIVA in Figure 4(a) identified 29 significant differences, the largest number among the algorithms. Most of these differences are in the AUD, MOT, and VIS connections, indicating possible biomarkers. In Figure 4(c), ar-cEBM detected 17 significant differences, most in the MOT, VIS, and CC connections. On the other hand, in Figure 4(b), ar-cIVA identified 10 significant differences, the fewest among the algorithms. Despite these variations in the number of detected differences, we observe commonalities in the MOT and AUD connections across all algorithms, indicating replicability of the sensitivity in these domains.

**FIGURE 5.**
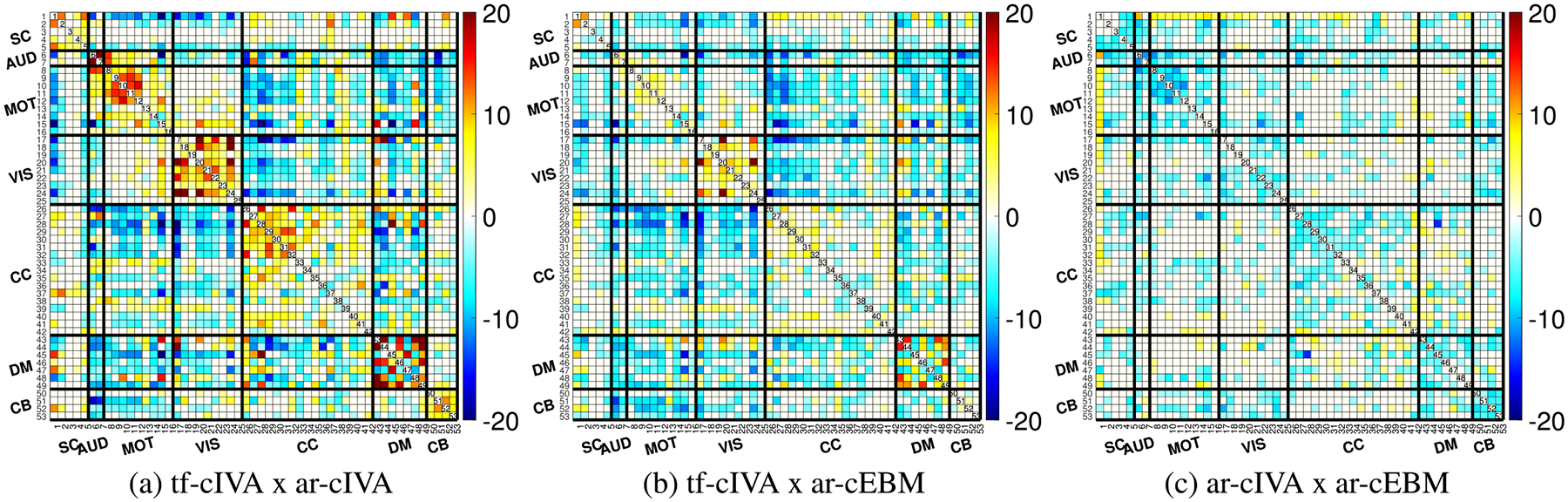
Paired t-test comparisons (p<0.05, FDR corrected) of the estimated temporal FNC matrices between algorithms. The panels show t-value maps for (a) tf-cIVA vs. ar-cIVA, (b) tf-cIVA vs. ar-cEBM, and (c) ar-cIVA vs. ar-cEBM. The most pronounced differences are observed when comparing tf-cIVA to the other two methods (a, b), reflecting its higher modularity. Fewer differences are seen between ar-cIVA and ar-cEBM (c), suggesting that their estimated temporal connectivity structures are more similar to each other.

To quantify the differences among the algorithms’ temporal FNC structure, we performed paired t-tests comparing the Fisher z-transformed FNC matrices [17]. This comparison identifies where the connectivity differs significantly between the two methods. The results, shown as t-value maps after FDR correction (p<0.05) in Figure 5, present substantial differences between all pairs of algorithms. Comparing the two IVA-based methods, tf-cIVA vs. ar-cIVA in Figure 5(a), we observe widespread significant differences across the FNC matrix, with positive values in the block diagonal and negative in the cross-domains area, reinforcing what we noticed previously with the mean temporal FNCs in Figure 3, where tf-cIVA presents a clearer block structure than ar-cIVA. In Figure 5(b), tf-cIVA vs. ar-cEBM shows fewer differences in the main block diagonal, but the negative values in the cross-domain blocks also show the most prominent difference between the algorithms, consistent with Figure 3. The comparison between ar-cIVA and ar-cEBM in Figure 5(c) shows fewer significant differences relative to the comparisons involving tf-cIVA, suggesting that the temporal FNCs from both algorithms are similar. This indicates a high degree of replicability between the two algorithms employing the adaptive-reverse scheme despite the differences in how they leverage the joint information. Overall, these pairwise comparisons demonstrate that the choice of constraint application (threshold-free vs. adaptivereverse) has a greater influence on the resulting temporal FNC estimates, with tf-cIVA producing a significantly more modular and clean temporal connectivity structure.

#### 2) SPATIAL FNCS

To complement the analysis, we investigated the spatial connectivity between the estimated brain networks using spatial FNCs. This metric provides information about the spatial map overlap of different networks. To calculate the spatial FNC, the spatial maps estimated by each algorithm were first converted to z-scores and then thresholded at |z|=2 to retain voxels showing high activation. Then, Pearson correlation coefficients were computed between these z-scored thresholded spatial maps for all pairs of components, resulting in a symmetric spatial FNC matrix. We also calculated the modularity of each matrix after thresholding to maintain a link density between 20 and 70%.

**FIGURE 6.**
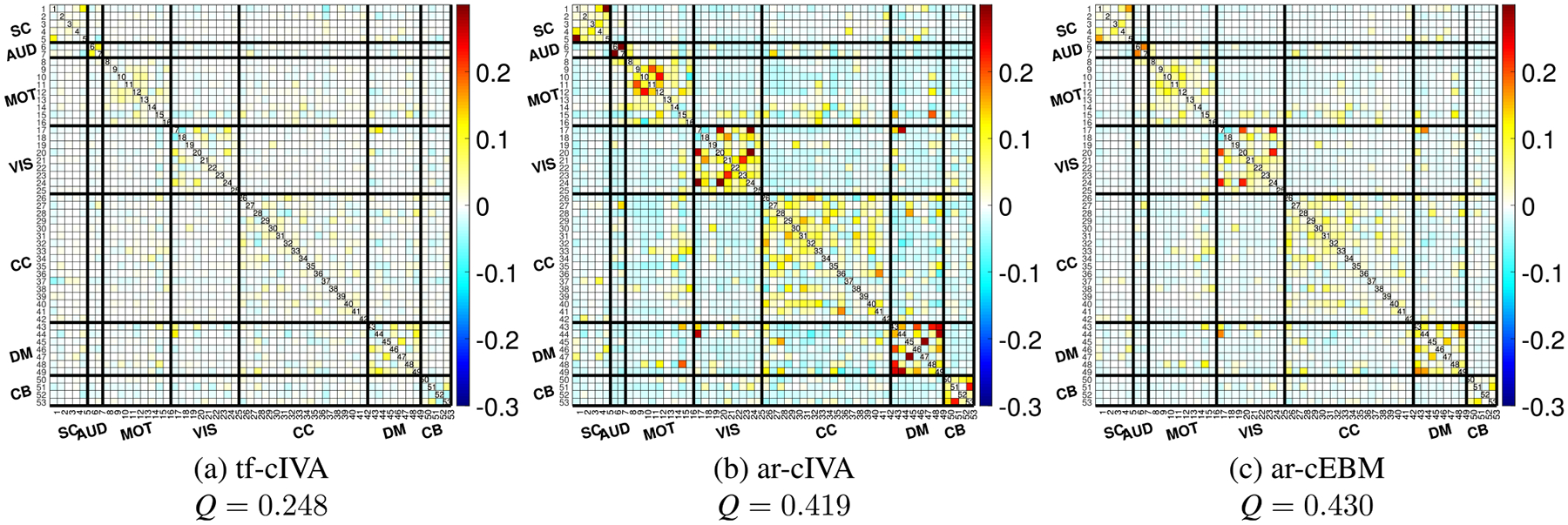
Mean spatial correlation FNC matrices, averaged across all subjects. Panels show results for (a) tf-cIVA, (b) ar-cIVA, and (c) ar-cEBM. Both ar-cIVA and ar-cEBM exhibit structured off-diagonal correlations, indicating spatial similarity between different networks. In contrast, tf-cIVA produces a highly diagonal matrix, an expected consequence of its cost function, which penalizes spatial overlap. This difference is also reflected in the higher spatial modularity values for ar-cIVA and ar-cEBM.

The mean spatial FNC matrices are shown in Figure 6. The results indicate differences in the estimated spatial structure. Both ar-cIVA in Figure 6(b) and ar-cEBM in Figure 6(c) produced matrices with noticeable block-diagonal positive correlations and anticorrelations in the cross-domains areas, which is reflected in their respective modularity values: 0.419 and 0.430. In contrast, the matrix produced by tf-cIVA in Figure 6(a) is almost diagonal, with off-diagonal correlations close to zero and resulting in the lowest modularity value among the spatial FNCs, Q=0.248. This outcome is likely a result of tf-cIVA’s cost function, which includes a term that penalizes spatial similarity between components and non-corresponding references.

**FIGURE 7.**
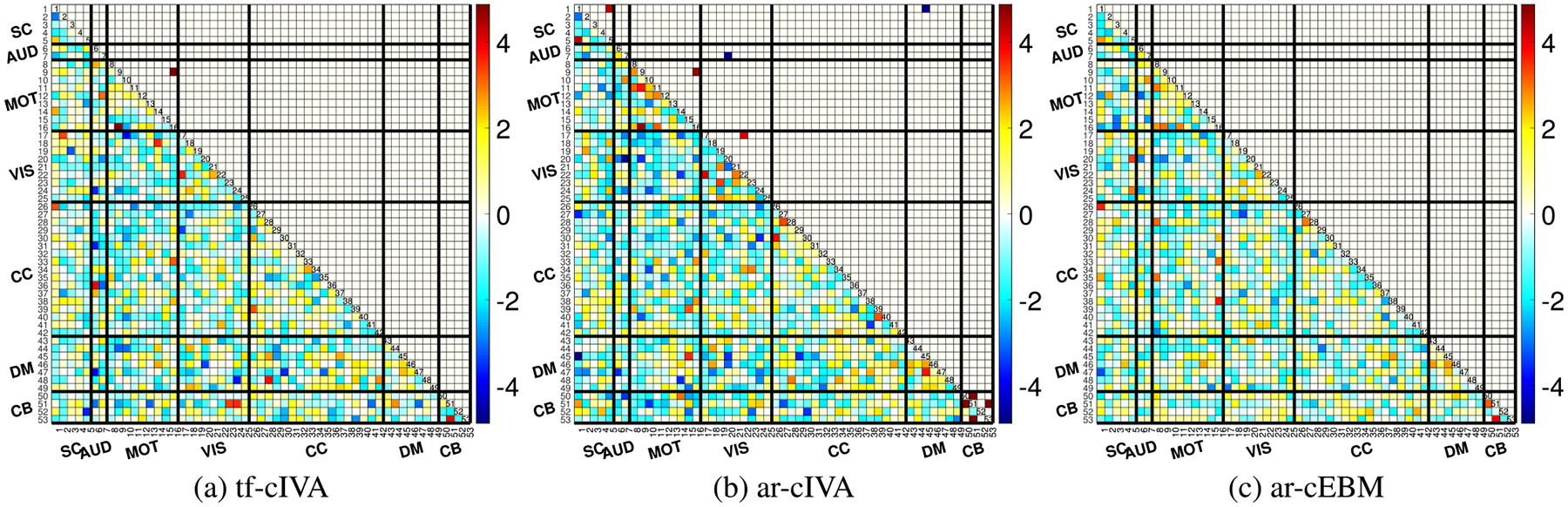
Two-sample t-test results (p<0.05, FDR corrected) for spatial FNCs comparing Healthy Controls (HC) and Schizophrenia (SZ) patients. Panels show results for (a) tf-cIVA, (b) ar-cIVA, and (c) ar-cEBM. The ar-cIVA algorithm (b) identifies significantly more group differences after FDR correction. In contrast, tf-cIVA (a) yields only one significant difference, whereas none of the ar-cEBM (c) values pass FDR correction. The results are summarized in Table 3.

We performed two-sample t-tests to compare the spatial FNC matrices between HC and SZ groups for each algorithm, applying FDR correction (p<0.05). The results are shown in Figure 7, where the lower diagonal shows all t-statistics and the upper diagonal shows the significant differences that survived FDR. After correction, ar-cIVA in Figure 7(b) identified 7 significant differences, compared with just 1 that tf-cIVA identified in Figure 7(a), and ar-cEBM identified no significant differences after correction in Figure 7(c) (Table 3). These results suggest ar-cIVA is best suited for identifying group-level differences in the spatial connectivity of brain networks.

**FIGURE 8.**
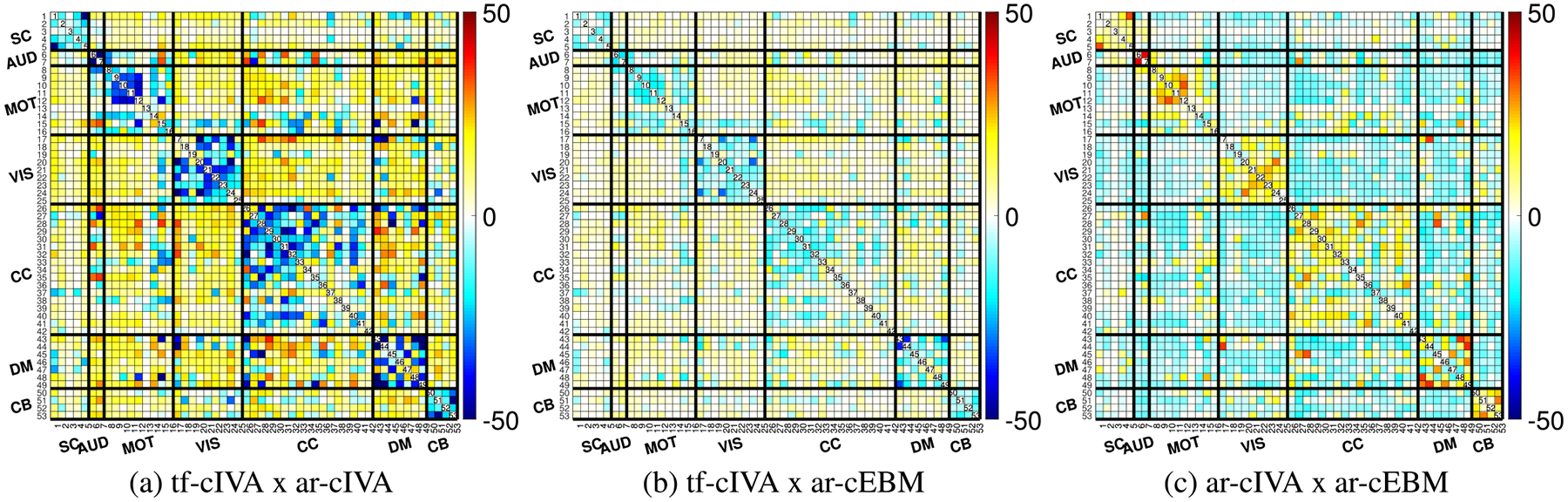
Paired t-test comparisons (p<0.05, FDR corrected) of the estimated spatial FNC matrices between algorithms. The panels compare (a) tf-cIVA vs. ar-cIVA, (b) tf-cIVA vs. ar-cEBM, and (c) ar-cIVA vs. ar-cEBM. The most substantial differences appear when comparing tf-cIVA to the other methods (a, b), reflecting its spatially decorrelated structure. In (c), we notice that ar-cIVA and ar-cEBM present a similar block structure, but the correlation values obtained by ar-cIVA are significantly higher.

Figure 8 shows the paired t-tests after FDR correction (p<0.05). The most substantial differences are observed when comparing tf-cIVA to the other two methods (vs. ar-cIVA in Figure 8(a) and vs. ar-cEBM in Figure 8(b)). These differences, particularly the strong negative t-values indicating higher block-diagonal correlations in the other methods, reflect the penalty for correlation with non-corresponding references in tf-cIVA’s cost function. The comparison between ar-cIVA and ar-cEBM in Figure 8(c) shows a block structure with positive block diagonal and negative cross-domain area, showing that both methods have a similar structure, but ar-cIVA correlation values are higher. These comparisons demonstrate that the different threshold handling approaches, especially the regularization used in tf-cIVA, lead to distinct estimations of spatial connectivity structure.

#### 3) MODULARITY AND FDR SUMMARY

Table 2 summarizes the modularity values for each FNC type. For temporal FNCs, tf-cIVA and ar-cEBM achieved higher modularity, while for spatial FNCs, ar-cEBM and ar-cIVA performed best. Table 3 shows the number of significant connections surviving FDR correction for group comparisons.

### F. ACTIVITY

**FIGURE 9.**
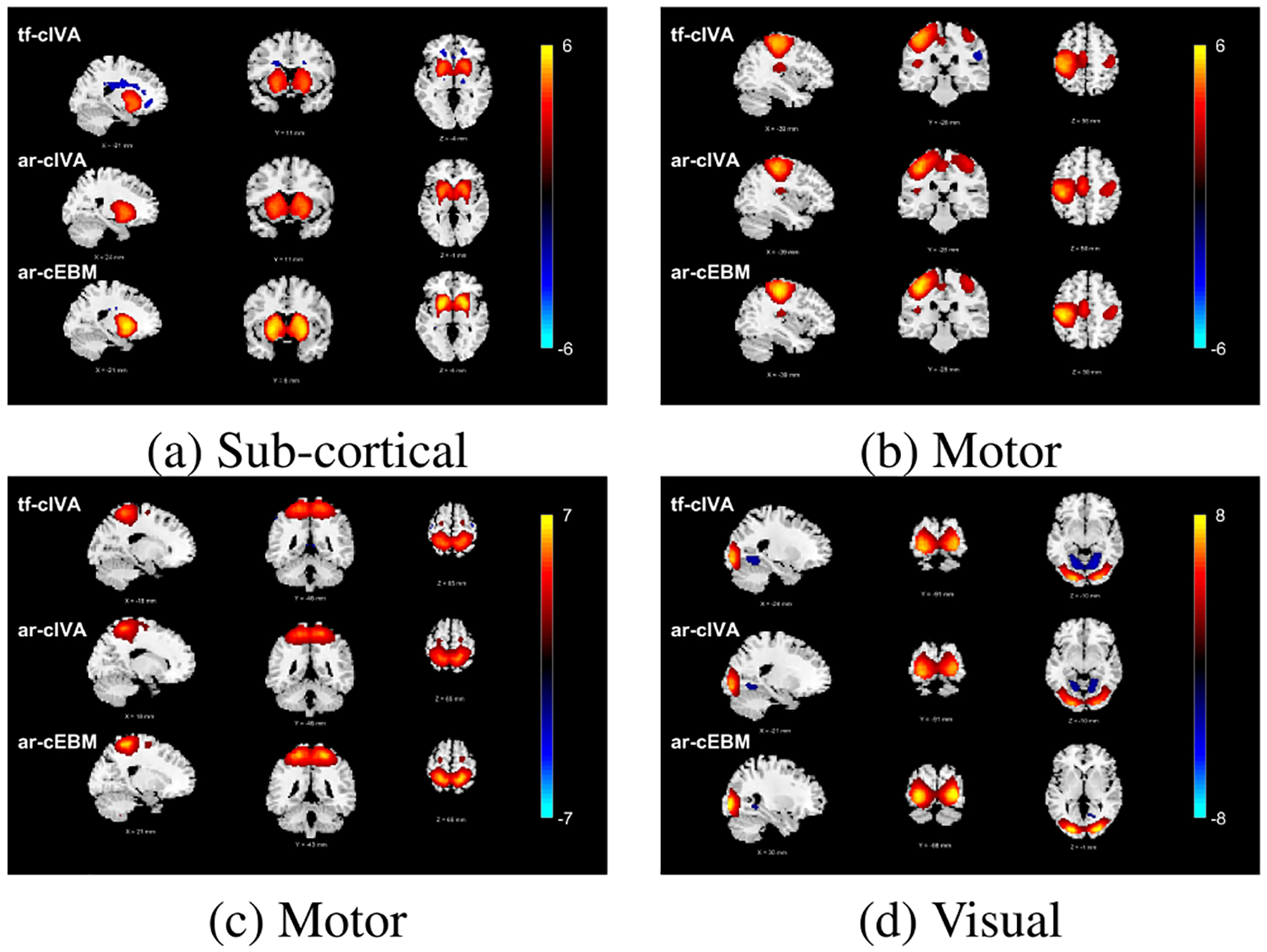
One-sample t-maps (p<0.05, FDR corrected) showing consistency across subjects for selected networks. For each component (a-d), the results for tf-cIVA, ar-cIVA, and ar-cEBM are displayed in successive rows. While all three algorithms successfully identify the expected patterns of brain activity, the t-maps from ar-cEBM consistently exhibit higher t-values. This indicates a higher degree of spatial consistency in the components estimated by the subject-wise ar-cEBM approach.

We evaluated the spatial consistency of the estimated brain networks across subjects using voxel-wise one-sample t-tests. Performing this test voxel-wise allows us to identify specific brain regions that show consistent activation across subjects, where higher absolute t-values indicate lower variability. For visualization purposes, the resulting group t-maps shown in Figure 9 were converted to z-scores and thresholded at |z|=2 to display the most consistent voxels. To quantitatively compare the overall consistency produced by each algorithm, in Table 4 we calculated the mean absolute t-statistic across all non-zero voxels and components at different thresholds.

As summarized in Table 4 and visually shown in Figure 9, ar-cEBM yields the highest mean t-statistic values for different thresholds. This indicates that, regardless of its subject-wise approach, the spatial maps estimated by ar-cEBM exhibit higher consistency compared with those estimated by ar-cIVA and tf-cIVA. This behavior may be explained by the flexible density matching of its formulation. Therefore, ar-cEBM efficiently leverages HOS and shows more robustness against noise or outliers, leading to more consistent components for each subject.

### G. COMPUTATIONAL COMPLEXITY AND SCALABILITY

A major challenge in multi-subject fMRI analysis is computational cost. The three algorithms evaluated in this study present some trade-offs in this regard [28]. The ar-cEBM algorithm processes each subject’s data independently. Consequently, its computational time is expected to scale linearly with the number of subjects, K, scaling as $𝒪\left(KVN^{2}\right)$ [27]. In contrast, the IVA-based algorithms, ar-cIVA and tf-cIVA, process all subjects’ data jointly. This approach requires computational and memory resources to scale polynomially with K, with memory requirements scaling as at least $𝒪\left(N^{4}K+N^{3}K^{2}+NK^{4}\right)$ [45].

To empirically assess these properties, we ran the algorithms on a server equipped with a 40-core Intel Xeon E5-2670 v2 CPU and 128 GB of RAM. We then compared the wall-clock time for the cohort of 116 subjects from the Chicago site on a single server. In this scenario, the serial execution of ar-cEBM took around 4 hours and 45 minutes. It was slower than both ar-cIVA (around 3 hours and 15 minutes) and tf-cIVA (2 hours and 41 minutes). This shows that the significant cumulative workload involved in performing 116 independent decompositions in sequence can exceed the time required for a single constrained IVA run. Furthermore, the cross-ISI analysis revealed that tf-cIVA yields the lowest cross-ISI values. This high reproducibility is a practical computational advantage, as fewer independent runs are required to ensure the reliability of the estimated components, thus reducing the overall computational burden.

However, it is important to note that the subject-wise structure of ar-cEBM allows for the workload to be easily parallelized across a computing cluster, where the total analysis time can be ideally reduced to the time required for just a single subject. The IVA methods lack this scalability. Besides, the computational complexity of ar-cEBM increases less than the IVA-based methods in terms of the number of subjects K, which makes a significant difference for thousands of subjects. Therefore, the empirical results indicate that while IVA-based methods can be effective on a single machine, ar-cEBM’s subject-wise approach offers good scalability when using parallel computing.

## IV. CONCLUSION

In this work, we conducted a multidimensional comparison of three constrained ICA/IVA algorithms: ar-cIVA, tf-cIVA, and ar-cEBM, in order to understand their advantages and trade-offs for analyzing multi-subject fMRI datasets. We analyzed the formulation of each method and how its approach affects relevant metrics in fMRI analysis. Our results show replicability across the three methods, considering their spatial correlation with the references and the identification of biomarkers. Besides these commonalities, each algorithm offers distinct strengths, highlighting clear trade-offs.

- **tf-cIVA** shows good performance regarding stability. It is the most reproducible algorithm across multiple random initializations, reducing the need for run selection and offering a computational advantage. It also produces the cleanest temporal FNCs with the highest modularity and shows good sensitivity in detecting group-level differences when analyzing the correlation of the components with their respective references. This algorithm can be useful in projects where the structure of temporal FNC (e.g., in dynamic analysis) or consistency is the primary interest.
- **ar-cIVA** demonstrates superior performance for spatial analysis. The algorithm achieves the highest spatial correlation with the reference templates and demonstrates superior sensitivity in detecting group-level differences in spatial FNCs. Therefore, this algorithm is the most compelling choice for studies focused on spatial connectivity biomarkers.
- **ar-cEBM** stands out because of its subject-wise approach, which can be efficiently scaled in multi-subject fMRI analysis through parallelization. This approach makes ar-cEBM the best-suited method for large-scale datasets (hundreds/thousands of subjects) due to its scalability. It also produces the most consistent spatial maps across subjects (highest t-statistics) and shows a balanced performance with good modularity in both temporal and spatial FNCs. Furthermore, it successfully identified significant group differences in temporal FNC.

**FIGURE 10.**
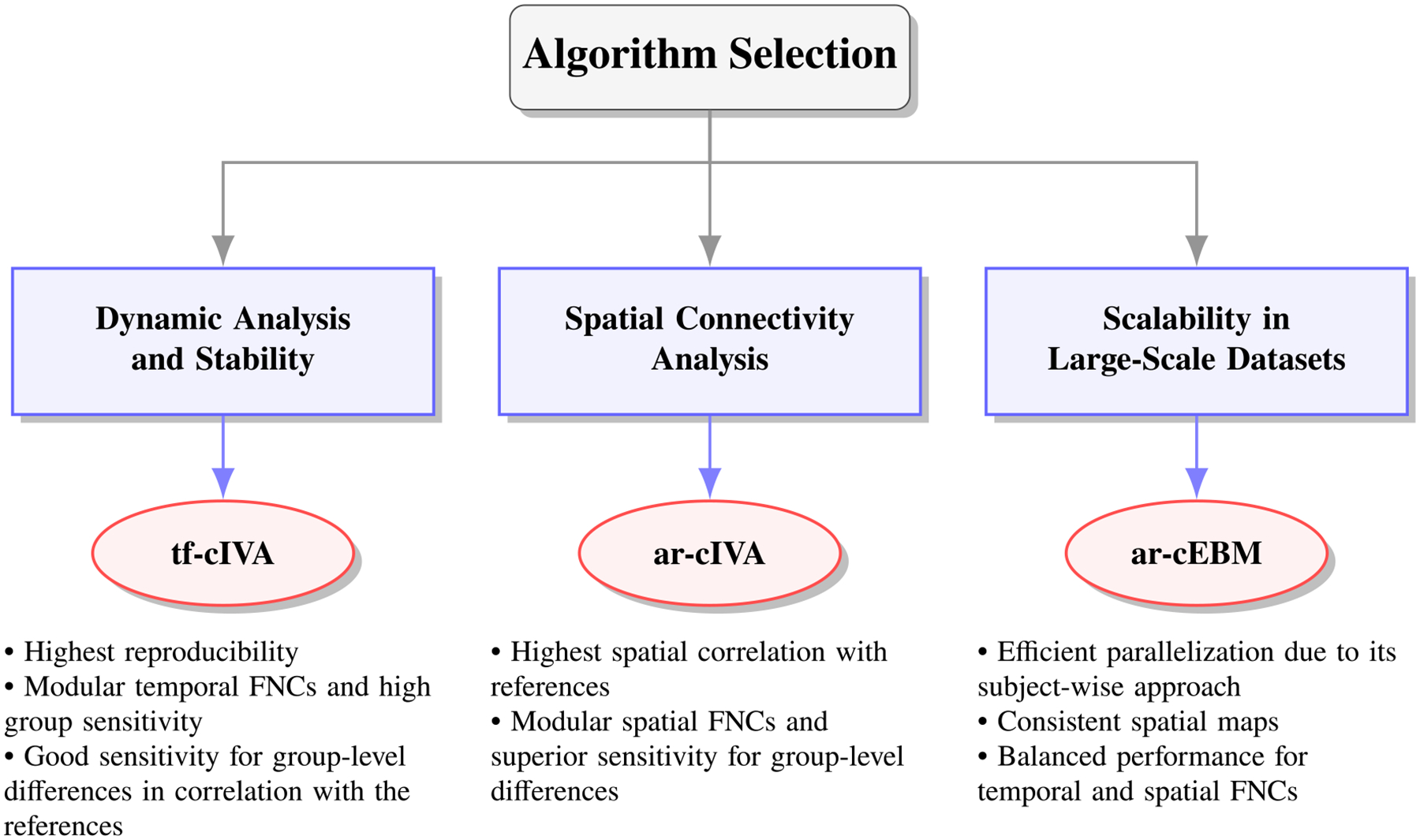
Decision schematic for selecting the appropriate algorithm according to different research goals.

Therefore, the choice of the method depends on the research goal, and choosing it appropriately can leverage the algorithm’s strengths to achieve better results. The recommendations are summarized in the Figure 10.

## Figures and Tables

**TABLE 1.** Reproducibility of each algorithm across 20 runs. Values represent the mean ± standard deviation for both the cross-joint ISI and the partial cross-joint ISI (calculated using the constrained components only). Lower values indicate higher stability. The results show that tf-cIVA is the most stable, followed by ar-cIVA and then ar-cEBM.

Algorithm	Partial Cross-ISI (10^−3^)	Cross-ISI (10^−3^)
tf-cIVA	**0.269** ± **0.006**	**57.674** ± **1.053**
ar-cIVA	21.722 ± 0.172	74.667 ± 2.458
ar-cEBM	33.364 ± 0.207	203.013 ± 0.524

**TABLE 2.** Modularity values for mean temporal and spatial FNC matrices. Modularity quantifies the degree to which a network can be subdivided into distinct, non-overlapping modules. Higher values indicate a more well-defined modular structure. The results show that tf-cIVA produces the most modular structure in temporal FNCs, while ar-cEBM and ar-cIVA yield more modular structure in spatial FNCs.

FNC Type	tf-cIVA	ar-cIVA	ar-cEBM
Temporal	**0.289**	0.154	**0.260**
Spatial	0.248	**0.419**	**0.430**

**TABLE 3.** Number of significant FNC differences between HC and SZ groups after FDR correction (p < 0.05). The table summarizes the sensitivity of each algorithm in detecting group differences for both temporal and spatial connectivity. The results show that tf-cIVA is the most sensitive to group differences in the temporal domain, while ar-cIVA is the most sensitive in the spatial domain.

FNC Type	tf-cIVA	ar-cIVA	ar-cEBM
Temporal	**29**	10	17
Spatial	1	**7**	0

**TABLE 4.** Mean t-statistic values from one-sample t-tests of the group spatial maps. The table reports the average t-value across all non-zero voxels and components at different thresholds. Higher mean t-values suggest greater spatial consistency across subjects. The results consistently show that ar-cEBM produces the most spatially similar group maps across all evaluated thresholds.

Threshold	tf-cIVA	ar-cIVA	ar-cEBM
0.5	**1.249**	1.175	**1.250**
1.0	1.898	1.842	**1.994**
1.5	2.542	2.521	**2.751**
2.0	3.146	3.069	**3.311**
2.5	3.645	3.531	**3.770**
3.0	4.072	3.994	**4.187**
